# Beneficial effects of the nutritional supplements on the development of diabetic retinopathy

**DOI:** 10.1186/1743-7075-11-8

**Published:** 2014-01-30

**Authors:** Renu A Kowluru, Qing Zhong, Julia M Santos, Mangayarkarasi Thandampallayam, Doug Putt, Dennis L Gierhart

**Affiliations:** 1 Department of Ophthalmology, Kresge Eye Institute, Detroit, MI, USA; 2 Zeavision, L.L.C., Chesterfield, MO, USA

**Keywords:** Carotenoids, Diabetic retinopathy, Macular pigment, Mitochondria, Nutritional supplements, Zeaxanthin

## Abstract

**Purpose:**

Increased oxidative stress and inflammatory mediators are implicated in the development of diabetic retinopathy, and in rats, its development can be prevented by antioxidants. Carotenoids are some of the powerful antioxidants, and diabetes decreases lutein and zeaxanthin levels in the serum and retina. The aim of this study is to investigate the effect of carotenoid containing nutritional supplements (Nutr), which is in clinical trials for ‘Diabetes Vision Function’, on diabetic retinopathy.

**Methods:**

Streptozotocin-induced diabetic rats (Wistar, male) were fed Purina 5001 supplemented with nutritional supplements containing zeaxanthin, lutein, lipoic acid, omega-3 fatty acids and other nutrients, or without any supplementation. Retinal function was analyzed at ~4 months of diabetes by electroretinography. After 11 months of diabetes, capillary cell apoptosis (TUNEL-staining) and histopathology (degenerative capillaries) were quantified in trypsin-digested retinal vasculature. Retina was also analyzed for mitochondrial damage (by quantifying gene expressions of mtDNA-encoded proteins of the electron transport chain), VEGF and inflammatory mediators, interleukin-1β and NF-*k*B.

**Results:**

Diabetes impaired retinal function decreasing the amplitudes of both a- and b-waves. In the same animals, retinal capillary cell apoptosis and degenerative capillaries were increased by 3–4 fold. Gene expressions of mtDNA encoded proteins were decreased, and VEGF, interleukin-1β and NF-*k*B levels were elevated. Supplementation with the nutrients prevented increased capillary cell apoptosis and vascular pathology, and ameliorated these diabetes-induced retinal abnormalities.

**Conclusions:**

Nutritional supplementation prevents diabetic retinopathy, and also maintains normal retinal function, mitochondrial homeostasis and inflammatory mediators. Thus, this supplementation could represent an achievable and inexpensive adjunct therapy to also inhibit retinopathy, a slow progressing disease feared most by diabetic patients.

## Introduction

Retinopathy remains one of the most devastating complications of diabetes. Despite major advances in basic science and clinical research to understand the complex pathophysiology of this blinding disease, the exact mechanism remains elusive and effective treatment modalities unclear. In diabetes, retina and its capillary cells experience increased oxidative stress, and their mitochondria become dysfunctional and mitochondrial DNA (mtDNA) is damaged [1-6]. In addition to capillary cells, increased oxidative stress is also observed in other retinal cells, including photoreceptors and retinal pigment epithelial cells [7,8]. Supplementation of antioxidants to diabetic rats containing lipoic acid, multi-antioxidants or AREDS-based antioxidants, prevents the onset of diabetic retinopathy [4,9,10].

In addition to oxidative stress, the retina also presents many abnormalities consistent with other inflammatory diseases, and animal models have provided promising results with anti-inflammatory therapies, suggesting that retinopathy is a low-grade chronic inflammatory disease [11,12]. Vascular endothelial growth factor (VEGF), an angiogenic factor important in vascular permeability and neovascularization, is elevated in the retina and vitreous of diabetic patients and animals, and this increase is associated with the manifestation of diabetic retinopathy [13-15]. Furthermore, redox-sensitive nuclear transcriptional factor-B, NF-*k*B, which is important in regulation of the expression of cytokines and growth factors, is activated, and the levels of pro-inflammatory mediators, such as interleukin-1β (IL-1β) and Intercellular Adhesion Molecule-1 (ICAM-1) are increased [16,17]. Rodent models have demonstrated that the antioxidants that inhibit the development of diabetic retinopathy, also inhibit increases in retinal NF-*k*B and IL-1β [17,18].

The high content of polyunsaturated fatty acids, combined with the highest oxygen uptake and glucose oxidation relative to any other tissue, makes the retina highly susceptible to oxidative stress [19,20]. However, to counteract the oxidative stress, carotenoids (organic pigments with antioxidant properties) are actively concentrated in the human eye [21]. Among this family of carotenoids, the macular pigments lutein, and zeaxanthin have been found to have important antioxidant and photoprotective activities [22,23]. Although lycopene and beta-carotene are effective quenchers of singlet oxygen in the plasma, lutein and zeaxanthin are the only carotenoids that accumulate in the retina. Epidemiologic studies have shown an inverse association between the levels of lutein and zeaxanthin in the eye and age related degenerative diseases such as macular degeneration and cataracts [24,25]. We have shown that zeaxanthin administration in diabetic rats prevents increase in retinal oxidative stress and proinflammatory cytokines, VEGF, ICAM-1 [26], and antioxidants containing vitamin C, vitamin E, β-carotene, N-acetyl cysteine and other micronutrients inhibit the development of diabetic retinopathy [4,24].

The goal of this study is to investigate the effect of the nutritional supplementation, which is in ‘Diabetes Vision Function’ clinical trials to improve the structure and function of the retina, on the development of diabetic retinopathy. Using rodent model of diabetic retinopathy, the effect of multi-component nutrients was investigated on the retinal capillary cell apoptosis, degenerative capillaries and cell function. To examine the effect of this supplement on amelioration of mitochondrial dysfunction and on inflammatory cytokines, gene expressions of mtDNA-encoded *cytochrome b* and *ND1*, and the levels of VEGF, IL-1β and NF-*k*B were quantified.

## Methods

Rats: Wistar rats (male, 200-225 g) were made diabetic with streptozotocin, and divided into two groups. Rats in group I received powder diet (Purina 5001) supplemented with multi-nutritional supplements containing carotenoids (Nutr). This EyePromise-DVS is specifically formulated to improve the structure and function of the retina, and is now being used for Diabetes Vision Function Supplement Study Clinical Trials (Gov Identifier: NCT01646047). Each kilogram of Nutr Purina diet contained Vitamin C (as ascorbic acid, 300 mg), Vitamin D3 (Cholecalciferol, 10,000 IU), Vitamin E (d-alpha tocopherol, 300 IU), Fish Oil EE70% (1.6 g), EPA (eicosapentaenoic acid, 650 mg), DHA (docosahexaenoic acid, 500 mg), Benfotiamine (1 g), α lipoic acid (750 mg), tocomin (200 mg), zeaxanthin (40 mg), lutein (20 mg) and proprietary blend containing 300 polygonium duspidatum SE (resveratrol), green tea, turmeric root (curcumoids), N-acetyl-cysteine, Pyconogenol^®^ Pine Bark, grape seed extract, coenzyme Q10 and zinc (2.65 g), and soybean oil. Rats in group II received Purina diet without any supplementation (Diab), and age matched normal rats were used as control (Nor). Average daily food consumption of the diabetic rats during the entire duration of the study (11 months) was ~50 g. The rats were sacrificed with CO2 ~11 months after initiation of the experiment. One eye was suspended in 10% formalin for trypsin digestion to prepare retinal microvasculature, and the retina from the other eye was isolated to quantify biochemical parameters as routinely performed in our laboratory [9,10]. A slice of the liver was removed to confirm the uptake of some of the major constituents by HPLC. Treatment of rats was carried out as per the guidelines of National Institute of Health principles of laboratory animal care and the Association for Research in Vision and Ophthalmology resolution on the use of animals in research, and the institutional guidelines.

### Retinal capillary cell apoptosis and histopathology

Retina was isolated from the formalin-fixed eye, and rinsed overnight with the running water. The microvasculature was isolated by incubating the retina with 3% crude trypsin (Invitrogen-Gibco, Grand Island, NY) containing 200 mM sodium fluoride for 45 to 70 minutes at 37°C, and the neuro-retinal tissue was gently brushed away. The apoptotic vascular cells were detected by incubating the preparation with terminal deoxyribonucleotide transferase (TdT)-mediated dUTP nick end labeling stain (TUNEL; In Situ Cell Death kit; Roche Molecular Biochemicals, Indianapolis, IN). In each experiment, a positive control was run by exposing the retinal vessels to DNAse before initiation of the TUNEL reaction [9,10]. TUNEL positive cells were identified in a masked fashion, and each trypsin digest was surveyed systematically under a Zeiss Axiophot photomicroscope.

After TUNEL staining, the microvasculature was stained with periodic acid-Schiff and hematoxylin for histologic evaluation. The number of acellular capillaries was counted in multiple mid-retinal fields (one field adjacent to each of the 5–7 retinal arterioles radiating out from the optic disc) and expressed as total acellular capillaries per retina [9,10].

### Functional assay

Retinal function was determined at ~4 months of diabetes in rats by measuring electroretinogram (ERG) responses using Ocuscience HMsERG system. The rats were dark-adapted overnight, anesthetized with ketamine and xylazine, and their pupils were dilated with 1.0% tropicamide, 1.0% cyclopentalate hydrochloride, and 2.5% phenylephrine hydrochloride. The rats were placed on a heating platform and body temperature was monitored by a rectal thermometer. The amplitudes of a- and b-waves were measured by placing a silver embedded thread eye electrode at the corneal surface through a thin layer of 1% methylcellulose. Needle electrodes placed in the tail and cheek served as ground and reference electrodes, respectively. A dark-adapted intensity-response series was recorded using a series of Ganzfeld flashes with intensities ranging from 100–25,000 mcd.s/m^2, and the amplitudes of a- wave (initial negative deflection, the trough of the a-wave) and b-wave (positive deflection, trough of the a-wave to the peak of the b-wave) were analyzed [27,28].

Reactive oxygen species (ROS) were quantified by fluorescence spectroscopy using 2′,7′- dichlorofluorescein diacetate (DCFDA; Sigma-Aldrich, St. Louis, MO). Protein (5-10 μg) was incubated in PBS with 2 μM of DCHFDA for 10 minutes and fluorescence was measured at 485 nm excitation and 530 nm emission wavelengths [29].

Antioxidant capacity of the retina was measured using an assay which is based on the ability of the sample to inhibit oxidation of 2,2′-Azino-di-[3-ethylbenzthiazoline sulfonate]^+^ (ABTS) by metmyoglobin. The amount of ABTS^+^ produced was measured by decrease in absorbance caused by the antioxidants in the sample cause. The samples were measured in duplicate [15].

Mitochondrial DNA damage was determined by mitochondrial genome-specific quantitative extended-length PCR with the GeneAmp XL PCR kit. Products were resolved on an agarose gel and relative amplification was quantified by normalizing the intensity of the long product to the short product (mtDNA = 13.4 kb/210 bp). To confirm mtDNA damage, gene expressions of mtDNA-encoded proteins of the electron transport chain complex I (*ND1 & ND6*) and complex III (*cytochrome b, Cytb*) were quantified by real time PCR using the methods routinely employed in our laboratory [6,29].

Quantification of VEGF, NF*-k*B and IL-1β: The amount of VEGF was quantified by an ELISA method using a kit from the R&D Systems, Minneapolis, MN, as routinely performed in our laboratory [15,26].

Activation of NF-kB was estimated by ELISA using a TransAM NF-kB kit from Active Motif (Carlsbad, CA) as previously reported by us [10,30]. The assay is based on the principle that only the active form of NF-*k*B in the sample binds to oligonucleotide containing NF-*k*B consensus site (5′-GGGACTTTCC–3′) that is immobilized onto the microtiter plate, and the primary antibody (against p65 subunit of NF-*k*B) is accessible to the activated NF-*k*B which is bound to its target DNA. The concentration of IL-1β in the retina was quantified by an ELISA method using a rat-specific kit from R&D Systems, as previously reported by us [31]. Each sample was analyzed in duplicate.

### Statistical analysis

Results are presented as mean ± SD, and were analyzed using Sigma Stat software. The Shapiro-Wilk test was used to test for normal distribution of the data, and the data that did not present normal distribution, Kruskal-Wallis test followed by Dunn’s test was applied. P value <0.05 was considered as statistically significant.

## Results

### Nutritional supplements containing carotenoids prevent accelerated capillary cell apoptosis and histopathology associated with diabetic retinopathy

**Figure 1:**
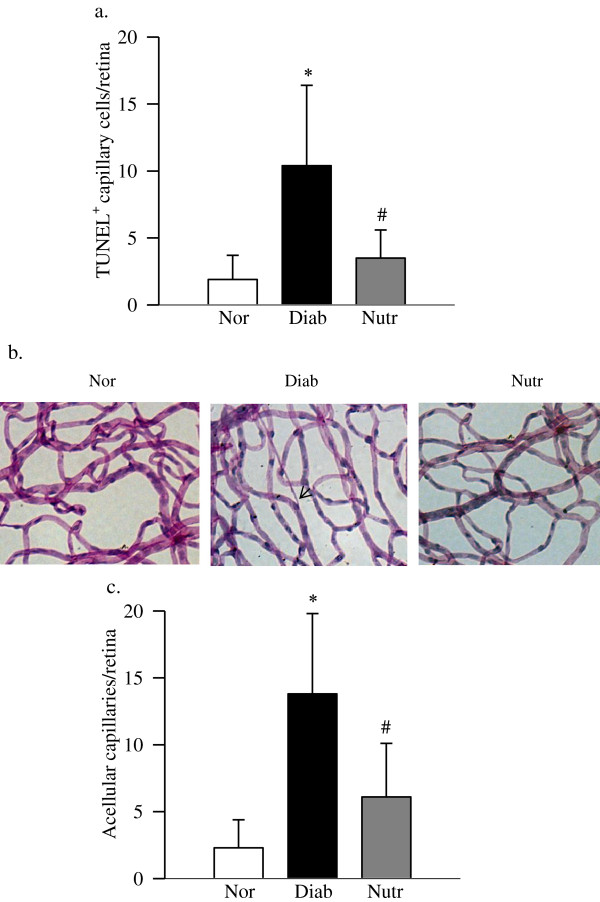
**Nutrient administration inhibits retinal capillary cell apoptosis and degeneration in diabetic rats.** Trypsin digested retinal microvasculature was **(a)** analyzed for capillary cell apoptosis by TUNEL staining. **(b)** After TUNEL staining, the microvessels were stained with periodic acid-Schiff-hematoxylin; the arrow indicates a capillary which has lost endothelial cell. **(c)** The number of acellular capillaries was counted in the entire retinal vasculature, and represented as number of acellular capillaries/retina. Results are expressed as mean ± SD of 7–8 rats each in normal (Nor), diabetic (Diab) and diabetic rats receiving the nutrients (Nutr) groups. *p < 0.05 compared to age-matched normal, and ^#^p < 0.05 compared to diabetes.

As expected, at ~11 months of diabetes in rats, the retinal vasculature had 3–4 fold increase in TUNEL-positive cells and degenerative capillaries. However, the nutritional supplements containing carotenoids ameliorated diabetes-induced increase in capillary cell apoptosis, the number of TUNEL-positive capillary cells was similar in diabetic rats treated with the nutrients and normal rats (Figure 1a). In the same Nutr-treated diabetic rats, the number of degenerative capillaries in the retinal vasculature was significantly decreased compared with the age-matched diabetic rats without any supplementation (Figure 1b). Figure 1c is included to show the quantification of the degenerative capillaries in the retina.

**Figure 2:**
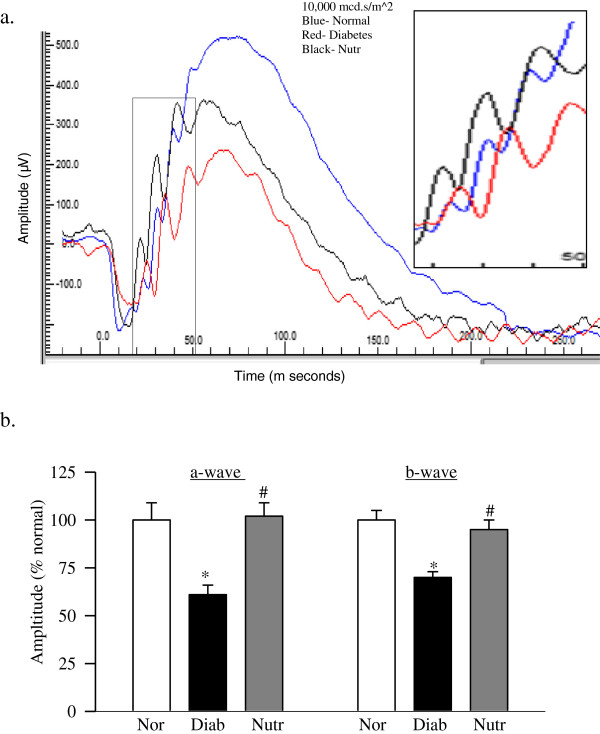
**Retinal dysfunction is ameliorated by the nutritional supplementation.** ERG was performed in dark-adapted rats at ~4 months of diabetes using Ocuscience HMsERG system. A dark-adapted intensity-response series was recorded using a series of Ganzfeld flashes with intensities ranging from 100–25,000 mcd.s/m^2. **(a)** Representative plots of a- and b-wave amplitudes at 10,000 mcd.s/m^2 are shown. The boxed portion is enlarged to show the delayed ERG response. **(b)** a-wave and b-wave amplitudes, obtained from normal rats are considered as 100%. The results are representative of 5 or more rats in each of the three groups (normal, diabetes and diabetes + Nutr). Blue = normal rats, Red = diabetic rats and Black = diabetic rats receiving the nutrients. *p < 0.05 compared to age-matched normal, and ^#^p < 0.05 compared to diabetes.

Retinal function was assessed by performing ERG measurements. Figure 2 shows significant decrease in the amplitudes of a- and b- waves at 10,000 mcd.s/m^2 in diabetic rats (>25%), and this was accompanied by delayed ERG response. Similar results were observed at 3,000 mcd.s/m^2 light fluxes (data not shown). These decreases in the amplitudes of a- and b- waves, however, were attenuated in diabetic rats receiving diet supplemented with Nutr (Figure 2b).

### Increase in retinal oxidative stress and mitochondrial damage are ameliorated in diabetic rats receiving the nutritional supplementation

**Figure 3:**
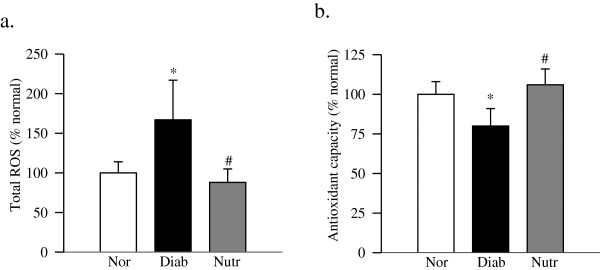
**Oxidative stress is ameliorated in diabetic rats receiving the supplementation. (a)** Total ROS levels were quantified in the retina (5-10 μg) using 2 μM of DCHFDA and the fluorescence emitted was measured at 485 nm and 530 nm. **(b)** Total antioxidant capacity of the retina was quantified by measuring the ability of the retina to inhibit oxidation of ABTS by metmyoglobin using 5-10 μg retina protein. Each sample was measured in duplicate, and the values are represented as mean ± SD of 6–8 rats in each group. *p < 0.05 and #p < 0.05 compared to normal and diabetes respectively.

Retinal ROS levels were significantly higher in diabetic rats, and the total antioxidant capacity, which includes the sum of antioxidant enzyme activities, macromolecules and small molecules, was significantly decreased compared with age-matched normal rats. However, diabetic rats receiving the supplement had significantly lower ROS levels and higher antioxidant capacity compared with diabetic rats without any supplementation (Figure 3a & 3b).

**Figure 4:**
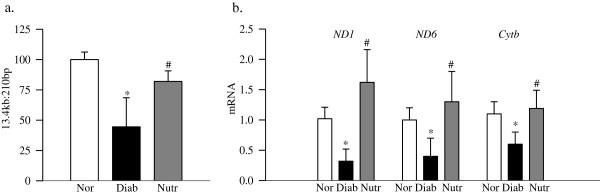
**The nutritional supplementation prevents diabetes-induced mitochondrial damage in the retina. (a)** Damage of mtDNA was evaluated by amplifying long (13.4 kb) and short (210 bp) regions by extended length PCR and conventional PCR respectively. **(b)** Gene expressions of *ND1, ND6 and Cytb* were quantified by real time PCR using *β-actin* as a housekeeping gene. Measurements were made at least in duplicate, and each value represents the mean ± SD from 6–8 rats/group. Nor = normal, Diab = Diabetes and Nutr = diabetic rats receiving the nutrients supplementation.*p < 0.05 compared to normal and ^#^p < 0.05 compared to diabetes.

In the same nutrient-supplemented diabetic rats, damage to mtDNA was also prevented, and this was further confirmed by significantly higher gene expressions of mtDNA-encoded proteins of the electron transport chain compared to the values from diabetic rats receiving no supplementation (Figure 4a & b). The levels of *ND1* and *ND6* of complex I and *cytb* of complex III in the retina were decreased by 40-80% in diabetic rats compared to normal rats, however, supplementation with the nutrients prevented such decreases, and the values obtained from the rats in the Nutr group were significantly higher compared to diabetic rats without any treatment.

### Administration of the nutritional supplements protects the retina from increase in inflammatory mediators

**Figure 5:**
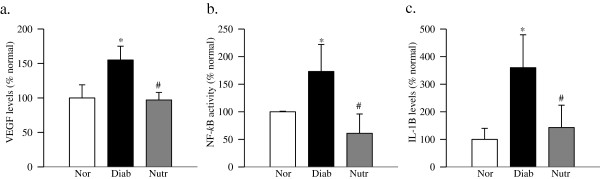
**Diabetes-induced increase in the retinal inflammatory cytokines is prevented in rats receiving nutritional supplementation.** Levels of **(a)** VEGF, **(b)** NF-*k*B and **(c)** IL-1β were quantified in the retina by using their specific ELISA kits. Each measurement was made in duplicate using retina from 5–6 rats in each of the four groups, and the values are represented as mean ± SD. *p < 0.05 and ^#^p < 0.05 compared to normal and diabetes respectively.

Diabetes in rats increased retinal VEGF levels by 50% compared to normal rats, and this increase in retinal VEGF was ameliorated by the nutrient supplementation (Figure 5a).

Since inflammation is also recognized as one of the critical drivers of diabetic retinopathy, we measured the effect of this nutritional supplementation on NF-*k*B, a redox-sensitive transcription factor which controls the expression of many genes involved with inflammation. As shown in Figure 5b, the supplementation with the nutrients protected diabetes-induced activation of retinal NF-*k*B, the values obtained from rats in the Nutr group were not different from those obtained from age-matched normal rats. Consistent with this, diabetes-induced increase in the levels of inflammatory cytokine IL-1β, was also ameliorated by the nutrients (Figure 5c).

### Supplementation with the nutrients does not ameliorate the severity of hyperglycemia in diabetic rats

Analysis of the liver samples for some of the major nutrients showed that the levels of α-tocopherol increased by 2 fold (36 μg/g to 74 μg/g), lutein by 3 fold (0.02 to 0.06 μg) and zeaxanthin by ~9 fold (0.008 to 0.07 μg/g) in the liver of diabetic rats receiving the nutritional supplements compared to diabetic rats without any supplementation. Our previous work has shown that the supplementation of rodent diet with 0.1% zeaxanthin increased zeaxanthin levels in the retina by almost 10 fold [26]. Collectively, this suggests that the regimen used in this study was successful in raising the levels of these nutrients in the retina. However, the severity of hyperglycemia, as measured by body weight, glycated hemoglobin (GHb) and average 24 hour urine volumes, was increased in diabetic rats compared with the normal rats, but, as shown in Table 1, the values obtained from the rats in Nutr group were not statistically different from those in diabetes group. This clearly implies that the beneficial effects of this nutritional supplementation on diabetes-induced retinal pathology and metabolism were not due to the amelioration in the severity of hyperglycemia.

**Table 1:** Effect of the nutritional supplementation on the severity of hyperglycemia

	**Body weight (g)**	**GHb (%)**	**Urine volume (ml/24 hours)**
Normal	618 ± 71	5.7 ± 0.6	9 ± 8
Diabetes	369 ± 65	11.1 ± 2.1*	106 ± 20*
Diab + Nutr	354 ± 63	9.8 ± 2.4*	92 ± 42*

Body weights were measured 2 times/week, GHb every 3 months, and 24 hours urine volumes every 15 days. The values are mean ± SD of 8–10 rats each in normal, diabetes and diabetes + Nutr groups. ^*^p < 0.02 compared to normal.

## Discussion

In the pathogenesis of diabetic retinopathy, accelerated apoptosis of retinal capillary cells precedes the formation of degenerative capillaries, and increased degenerative capillaries represent one of the features of retinopathy seen in diabetic rodents [32,33]. Our previous work has shown that the supplementation with antioxidants containing β-carotene in rodent model of diabetic retinopathy protects the retina from the development of degenerative capillaries. Furthermore, we have shown that AREDS-based antioxidants also protect the retina from pathology associated with diabetic retinopathy [9]. Carotenoids are not synthesized in animals, but are generally obtained from the diet. Among this group, α-, β- and γ-carotene, lycopene, lutein and zeaxanthin are some of the most abundant carotenoids in the North American diet, and luetin and zeaxanthin preferentially concentrate in the retina [21]. Diabetes is shown to decrease the macular pigment optical density and also retinal zeaxanthin levels [22,26]. Here, we show that administration of micronutrients containing carotenoids prevents accelerated loss of retinal capillary cells and the onset of diabetic retinopathy in a rodent model. This is accompanied by protection of the retinal dysfunction, which precedes the capillary cell apoptosis. This nutritional supplementation reduces oxidative stress and damage to the retinal mitochondria, and regulates VEGF and inflammatory mediators increased in diabetes. Thus, the supplementation, which is now being tested for diabetes-related visual dysfunction, appears to have potential to inhibit the development of diabetic retinopathy.

In diabetic retinopathy, increase in capillary cell apoptosis is considered as a surrogate marker which precedes the pathology characteristic of diabetic retinopathy [32]. Data presented here show that the nutritional supplementation protects the retina from both accelerated apoptosis of retinal capillary cells and from the formation of degenerative capillaries.

Although the pathology associated with diabetic retinopathy is observed in the retinal vasculature, several physiologic and functional abnormalities are observed before this histopathology appears. These functional abnormalities are mainly neuronal in origin; and neuronal cell apoptosis can be seen as early as one month after induction of diabetes [34,35]. Abnormal ERG responses appear before vascular lesions begin to appear; streptozotocin-induced diabetic rats present delayed ERG responses within one month of induction of diabetes [36], while retinal capillary cell apoptosis and histopathology are not observed till the duration is extended to at least 6 months [32]. Similarly, alterations in multifocal ERG are considered to predict the onset and progression of retinopathy in diabetic patients [37,38]. Furthermore, recent studies have implicated photoreceptors in the development of early stages of diabetic retinopathy, and the possible mechanism appears to be increase in oxidative stress [8]. The results presented here clearly show that the supplementation with the nutrients, in addition to protecting the retina from vascular abnormalities, also helps in the neuronal function as demonstrated by amelioration of deficits in the amplitudes of both a- and b- waves. These results demonstrate that this supplementation, in addition to protecting the retinal vasculature, also protects the non-vascular cells, including photoreceptors, bipolar, amacrine and Muller cells. Thus, this nutritional supplementation has potential to inhibit abnormalities associated with the early stages of diabetic retinopathy before the capillary cells of the retina begin to die and histopathology starts to appear.

Diabetes increases oxidative stress in the retina and its capillary cells, and impairs the antioxidant defense mechanism. Mitochondrial superoxide radicals are increased, and mitochondria become dysfunctional, their copy numbers are decreased and mtDNA is damaged. The damaged mtDNA results in decreased mtDNA-encoded proteins important in the electron transport chain, and this initiates a continuous cycle of free radicals [1-6]. Carotenoids are powerful antioxidants, and by scavenging free radicals, they protect the cells from the damage caused by free radicals [39,40]. Antioxidant properties of carotenoids are routinely linked with their beneficial effects on chronic diseases including diabetes, and the uptake of lutein and zeaxanthin in the retina is decreased in diabetes [22]. Furthermore, diets rich in carotenoids have shown protective effects against some of the chronic eye diseases, including age-related macular degeneration [21-23]. Our results suggest that the nutrient supplementation containing carotenoid prevents the development of diabetic retinopathy by protecting mtDNA from undergoing damage, and thus preventing the initiation of the self-propagating cycle. In support, dietary wolfberry supplementation (which contains zeaxanthin) has been shown to protect from decrease in TFAM and mitochondria copy number [41,42], which the retina experiences in diabetes [29,43].

VEGF, a hypoxia-induced factor is considered as one of the major growth factors in the development of diabetic retinopathy [13,14]; Diabetes-induced increase in VEGF plays a pivotal role in the increased cell permeability during the early stages of diabetic retinopathy, and in later stages of the disease VEGF is implicated in the angiogenesis. Antioxidants administration protects the retina from increases in VEGF [44], and we have shown that zeaxanthin or curcumin supplementation for 2 months in diabetic rats attenuates increase in retinal VEGF [15]. Here, we provide data showing that the long-term administration is also protective, retinal VEGF remains normal in diabetic rats treated with the nutrients. This clearly implies that the carotenoids protect increase in VEGF, both in the early stages of the disease, and also during the later stages of diabetic retinopathy when the capillary cells are being lost and the capillaries are degenerating.

Diabetic retinopathy is also considered as a low-grade chronic inflammatory disease, and sub-clinical inflammation is responsible for many of the vascular lesions seen in patients with diabetic retinopathy. The levels of inflammatory mediators (NF-*k*B, IL-1β*,* tumor necrosis factor α, and ICAM-1 etc.) are elevated in the retina in diabetes and leukostasis is increased [11,12]. NF-*k*B, a redox-sensitive transcriptional factor, controls the transcription of DNA, and also plays a central role in activating pro-inflammatory genes. Diabetes activates NF-*k*B in the retina and its vascular cells, and in the pathogenesis of retinopathy, activation of NF-*k*B is considered to act as pro-apoptotic [18,45]. Furthermore, activation of NF-*k*B elevates IL-1β, and IL-1β plays an important role in retinal capillary cell death and the formation of acellular capillaries, the microvascular pathology that is characteristic of retinopathy in diabetes, and the antioxidants, which inhibit the development of diabetic retinopathy in rodent models, also inhibit diabetes-induced increases in retinal IL-1β [17,31,46]. We have shown that one of the key events via which inflammation could contribute to the activation of the apoptotic machinery resulting in the development of diabetic retinopathy, could be the damage to the mitochondria [17,31]. Here, we show that the this supplementation also prevents diabetes-induced activation of NF-*k*B in the retina. These data further strengthen the hypothesis that the beneficial effects of the nutrients on diabetic retinopathy are mediated via inhibition of both inflammation and mitochondrial damage. However, with the encouraging results of these nutrients on inflammatory mediators and VEGF, the possibility that this supplementation also protects blood-retina barrier, an abnormality which can be seen during early stages of diabetic retinopathy [47], cannot be ruled out.

In summary, our data demonstrate that the nutritional supplementation, which is now in preclinical trials for maintaining the structure and function of the retina of human subjects with long term diabetes, also protects neuronal cells and vascular cells, and inhibits the development of retinopathy. This is achieved, possibly, via ameliorating increase in inflammatory mediators and maintaining mitochondria homeostasis, thus protecting the retina from the self-propagating vicious cycle of mitochondrial damage. Along with other experimental data demonstrating the beneficial effects of strategies to ameliorate oxidative stress, and prevent/retard diabetic retinopathy [9,10,15,26], supplementation with this micronutrients appears as an inexpensive adjunct therapy to inhibit retinal dysfunction, and the onset of this blinding disease. We believe that with the shortcomings of the clinical studies presenting inconclusive results with the antioxidants [48,49], results from a controlled clinical trial with this supplementation, which is already being tested for preserving retinal structural and functional abnormalities associated with diabetes, could help diabetic patients inhibit retinopathy, and spare them from losing their vision.

## Competing interest

RAK, QZ, JMS, MT and DP declare that they have no competing interests. DG is an investor in Zeavision, Chesterfield, and has a comercial interest.

## Authors’ contribution

RAK planned the experiments, performed the literature search, and wrote and edited the manuscript; QZ, JMS and MT performed the literature search and researched data; DP reserached data, and DJ planned the experiments and performed the literature search. RAK is the guarantor of this work and, as such, had full access to all the data in the study and takes responsibility for the integrity of the data and the accuracy of the data analysis. All authors read and approved the final manuscript.

## Authors’ information

Dr. Dennis Gierhart is an investor in Zeavision, Chesterfield, MO, and has a commercial interest.
