# Correction to: Beneficial effects of the nutritional supplements on the development of diabetic retinopathy

**DOI:** 10.1186/s12986-021-00620-w

**Published:** 2022-01-05

**Authors:** Renu A. Kowluru, Qing Zhong, Julia M. Santos, Mangayarkarasi Thandampallayam, Doug Putt, Dennis L. Gierhart

**Affiliations:** 1 Department of Ophthalmology, Kresge Eye Institute, Detroit, MI USA; 2 Zeavision, L.L.C., Chesterfield, MO USA

## Correction to Nutrition & Metabolism 2014,11:8 http://www.nutritionandmetabolism.com/content/11/1/8

Following the publication of the original article [1], the authors identified an error in Fig. 1. The correct figure is given below.

**Fig. 1:**
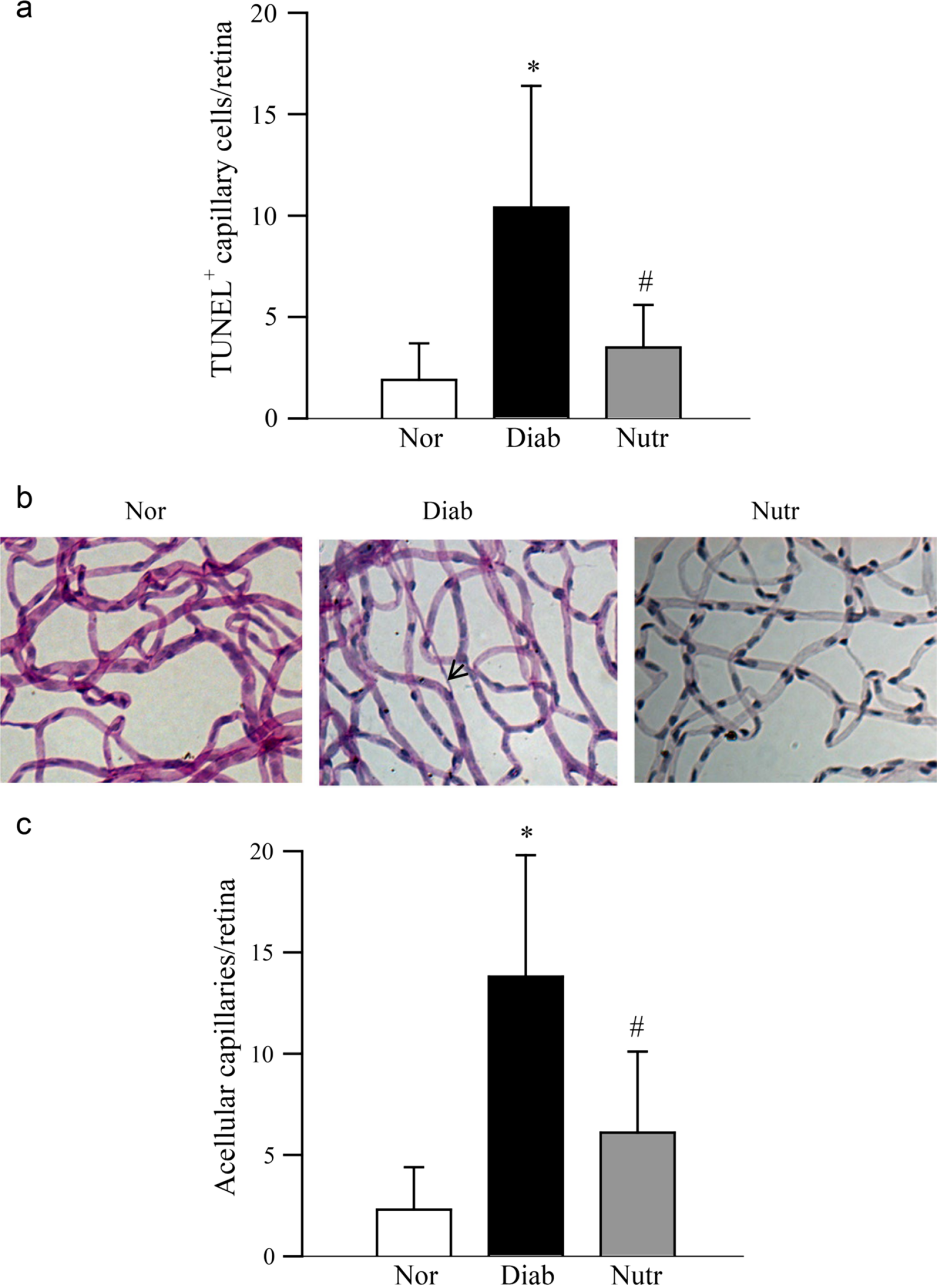
Nutrient administration inhibits retinal capillary cell apoptosis and degeneration in diabetic rats. Trypsin digested retinal microvasculature was (**a**) analyzed for capillary cell apoptosis by TUNEL staining. (**b**) After TUNEL staining, the microvessels were stained with periodic acid-Schiff-hematoxylin; the arrow indicates a capillary which has lost endothelial cell. (**c**) The number of acellular capillaries was counted in the entire retinal vasculature, and represented as number of acellular capillaries/retina. Results are expressed as mean ± SD of 7–8 rats each in normal (Nor), diabetic (Diab) and diabetic rats receiving the nutrients (Nutr) groups. *p < 0.05 compared to age-matched normal, and ^#^p < 0.05 compared to diabetes

The original article [1] has been corrected.
